# Relative Contributions of Land Use and Climate Change to Water Supply Variations over Yellow River Source Area in Tibetan Plateau during the Past Three Decades

**DOI:** 10.1371/journal.pone.0123793

**Published:** 2015-04-23

**Authors:** Tao Pan, Shaohong Wu, Yujie Liu

**Affiliations:** 1 Key Laboratory of Land Surface Pattern and Simulation, Institute of Geographic Sciences and Natural Resources Research, Chinese Academy of Sciences, Beijing 100101, China; 2 Center for System Integration and Sustainability, Michigan State University, East Lansing, MI 48823, USA; University of Aveiro, PORTUGAL

## Abstract

There is increasing evidence of environmental change impacts on ecosystem processes and services, yet poor understanding of the relative contributions of land use and climate change to ecosystem services variations. Based on detailed meteorological, hydrological records and satellite data over the Yellow River Source Area (YRSA) in Tibetan Plateau from 1980s to 2008, together with a water-yield module of Integrated Valuation of Ecosystem Services and Tradeoffs (InVEST) model and also a Residual Trends (RESTREND) method, we assessed the water supply variations in YRSA during the past three decades and disentangled the relative contributions of land use and climate change. Results show that water supply significantly decreased from 1980 to 2005 and then increased from 2005 to 2008. The quantity slightly decreased from 283.01mm in 1980 to 276.95mm in 1995, 270.12mm in 2000 and 267.97mm in 2005, and it then rebounded slightly to 275.26mm in 2008. The water supply variation ranged from 283.01mm to 267.97mm. Climate change contributed dominantly to water supply decrease from 1980 to 1995, which accounts for approximately 64% of the decrease. During 1995 to 2000, land use contributed more and about 58% to the water supply decrease as the intense human activities. From 2000 to 2005, climate change became a positive contribution to the water supply as the increased precipitation, but the land use still contributed negatively. From 2005 to 2008, both climate and land use have positive impacts, but land use contributed about 61% to the water supply increase. The implementation of the Three Rivers Source Area Ecological Protection Project has greatly improved the vegetation coverage conditions and the water retention ability during this period. We recommend that the implementation of ecological projects, grazing policies and artificial improvement of degraded grassland would help to conserve the water retention ability and increase water supply.

## Introduction

As persistent global climate and land use change continue to enhance the level of interference in ecosystems, the ecosystem structures and functions have been severely affected which degrades ecosystem services [1]. The degradation of ecosystem services not only impacts current human well-being but also will greatly reduce the ecosystem services providing for future generations [2–4]. Changes of ecosystems and their services are due to multiple interacting direct drivers (e.g., land use and climate changes), which in turn are controlled by indirect drivers (e.g., demographic, economic or cultural changes) [3]. Climate change is likely to affect water supply, carbon sequestration, critical habitats for biodiversity and many other ecosystem services by affecting terrestrial ecosystem abundance, production, distribution and quality [5–6]. Land use change can directly alter underlying surface conditions and properties as well as ecosystem types, which will further change the ecosystem structure and function as well as affect the ecosystem services [7–9].

Although many researchers have investigated the impacts of land use and climate changes on ecosystem services [10–14], a still existing key gap within current studies is that ecosystem services variations are often broadly attributed to land use and climate change. It is hard to distinguish the individual contribution as the effects due to land use and climate change are often considered together. As a result, adapting to climate and land use changes as well as effective ecosystem management will be difficult in optimally targeting mechanism of ecosystem services variation [15–16]. Hence, investigations into the relative impacts of land use and climate change on ecosystem services are very important for policy making on effectively climate change adaptation, ecosystem restoration and management, and also for optimal land use management [17–20].

The Yellow River is the second longest river in China which is called as the Mother River of China. As the region of the Yellow River source, the ecosystems of Yellow River Source Area (YRSA) has important water source recharging function which determine the water supply of the river. However, the water supply is vulnerable to average and extreme variations of land use and climate change [21–26]. The main ecosystem dominated by alpine meadow has been significantly degraded in recent years, especially the past three decades [27–28]. From the early 1990s to 2004, the grassland degradation area for the river source area reached 8.41×10^6^ hm^2^, accounting for 36.12% of the grassland area. Such degradation is significant for the YRSA and seriously affects the maintenance of the area’s water retention ability [29]. The observed records gathered in recent years at hydrological stations in river source areas indicate that the river runoff typically exhibits a downward trend [30]. Although many studies have been previously reported in the literature, conflicting views were found about the cause of this phenomenon. For instance, Zhang et al [30] suggest that climate change is the primary cause of runoff changes. However, Wang et al [31] believe that ecosystem degradation due to human activity is the primary reason for the reduced runoff as the ecosystem services were degraded. Which is the main driver of water supply variations in YASA, land use or climate change? What are the relative contributions of land use and climate change to water supply during the past three decades? These critical scientific questions are remaining unanswered. Only by addressing these questions can we use niche-targeting construction for ecological restoration.

In this study, based on the observed meteorological and satellite data from 1980s to 2008, and also the hydrological stations records in YASA, together with an integrated ecosystem service model, we aim to (1) investigate the spatial and temporal variations of the water supply variations; and (2) disentangle the relative contributions of climate and land use change to water supply, during the past three decades in YRSA.

## Materials and Methods

### Study area

**Fig 1:**
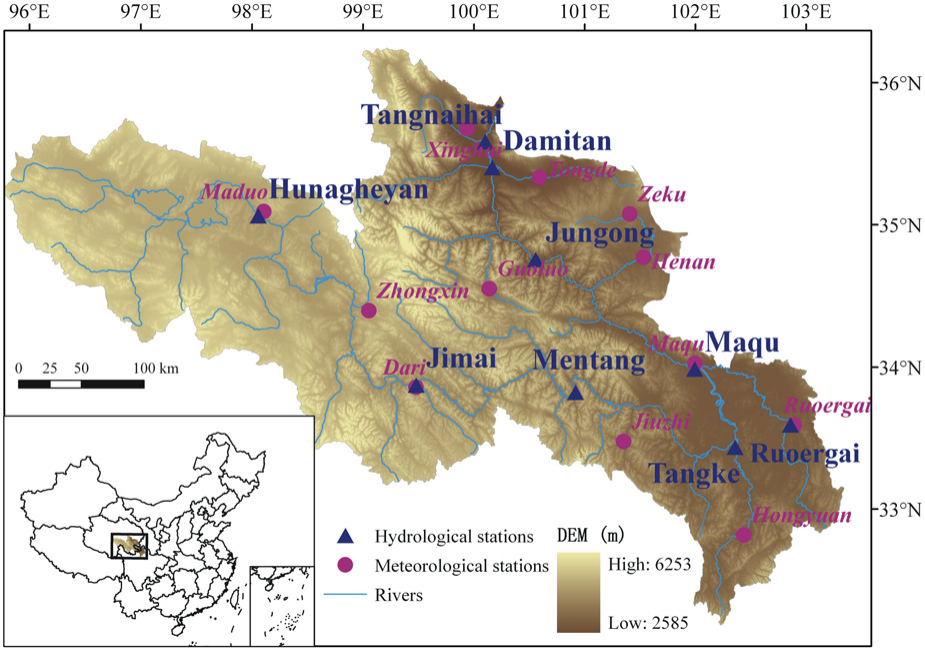
Location of the study area and the distribution of meteorological and hydrological stations, the resolution of the DEM is 90×90m.

The YRSA refers to the basin located between Yellow River Headwaters and Tangnaihai hydrological station. It lies in the northeastern of Tibetan Plateau between 95°50′-103°30′E and 32°20′-36°10′N (Fig 1). YRSA covers a catchment area of 121,972 km^2^ which is 16% of the Yellow River basin with average elevation above 3,000 m. The annual average temperature here is below zero, and the average precipitation is 485.9 mm within 1956 and 2010. The annual average runoff is 20.52 billion m^3^, which occupy 38% of the total runoff in Yellow River. So the YRSA is also called as the water tower of Yellow River. However, significant reduction of surface runoff, glacier area, number of lakes and frozen earth has been observed since 1990. The vegetation is getting degraded after the 1970s. The degraded pasture has increased by 30,000~75,000 hm^2^ every year, and the degradation speed in the 1990s is twice of that in the 1980s. There are 2.13 million hm^2^ dunes and black land so far in this area because of grassland degradation, which has led to some farmers’ resettlement.

### Data sources and processing

The YRSA land use and land cover datasets for 1980, 1995, 2000, 2005 and 2008 with 1×1 km^2^ pixel were extracted and interpreted from Landsat thermic mapper/enhanced thermic mapper (TM/ETM) satellite images. 26 TM/ETM images were selected for each one period. We used 1:100,000 topographic maps for geo-referencing. A minimum of 30 evenly distributed sites were selected as ground control points (GCPs) for each TM/ETM image. The land cover was classified into 9 types (Table 1). The accuracy assessment was performed for the classified maps of all five time steps. Stratified random sampling design was adopted for the accuracy assessment. The overall interpretation accuracy in the identification of land use and LULC categories from TM/ETM data is about 91%.

**Table 1:** Land cover types classification system for YRSA.

Types	Definition
**Arable land**	Land used primarily for production of food and fiber, including cultivated land, newly reclaimed wasteland, fallow land, swidden land, rotation land of grass, and also croplands for fruits, mulberry, agroforestry; also beaches and mudflats for cultivating more than three years.
**Forested land**	Forest lands for growing trees, shrubs, bamboo, as well as coastal mangrove forests and so on.
**High coverage grassland**	Natural grassland, improved grassland and mowing grassland that the coverage is higher than 50%. Such grasslands generally have good water conditions and grow well.
**Middle coverage grassland**	Natural grassland and improved grassland that the coverage is between 20% and 50%. Such grasslands generally have worse water conditions and grow relative sparsely.
**Low coverage grassland**	Natural grassland that the coverage is between 5% and 20%. Such grasslands generally lack of water and grow sparsely. The livestock using conditions is bad.
**Water area**	Natural terrestrial waters and water conservancy facilities lands.
**Settlements and other Construction land**	Urban and rural settlements, and mining, transportation and other land outside the towns.
**Unused land**	Unused lands including lands hard to use.

Climatic data of twelve meteorological stations were collected from the China Metrological Administration (CMA) including the daily maximum and minimum air temperatures, mean wind speed, sunshine hours, air pressure and relative humidity (Table 2). Version 4.2 of ANUSPLIN model was selected to achieve the spatialization of various meteorological elements. This model is a set of FORTRAN program package extended and developed by Australian National University on a basis of plate smoothing spline theory [32–33]. Potential evapotranspiration for the 12 stations was calculated using modified Penman-Monteith equation [34].

**Table 2:** Meteorological Stations in the Yellow River Source Area.

Number	Station	Province	Latitude (°N)	Longitude (°E)	Elevation (m)	Annual Mean Temperature (°C)	Annual Mean Precipitation (mm)
52943	Xinghai	Qinghai	35.58	99.98	3323.20	1.7	371.8
52957	Tongde	Qinghai	35.27	100.65	3289.40	0.7	430.2
52968	Zeku	Qinghai	35.03	101.47	3662.80	-1.6	510.5
56033	Maduo	Qinghai	34.92	98.22	4272.30	-3.4	328.2
56041	Zhongxin	Qinghai	34.27	99.2	4211.10	-3.9	471.6
56043	Guoluo	Qinghai	34.47	100.25	3719.00	-0.4	512.9
56046	Dari	Qinghai	33.75	99.65	3967.50	-0.7	560.4
56065	Henan	Qinghai	34.73	101.6	8500.00	-0.2	565.9
56067	Jiuzhi	Qinghai	33.43	101.48	3628.50	0.9	739.4
56074	Maqu	Gansu	34.00	102.08	3471.40	1.7	596.0
56079	Ruoergai	Sichuan	33.58	102.97	3439.60	1.4	637.9
56173	Hongyuan	Sichuan	32.80	102.55	3491.60	1.7	743.7

The hydrological data were collected from local hydrological bureaus (Table 3). A digital elevation model (DEM) data with 90m resolution from SRTM Digital Elevation Database was used to analyze the slope length/angle factors for sediment control. The 1:1000,000 soil data were collected from the Institute of Soil Science, Chinese Academy of Sciences, including the soil texture descriptions, soil depths and soil types. The YRSA was separated into 168 sub-watersheds based on the 90m resolution DEM using the hydrological analysis tools provided by ArcGIS 10.1.

**Table 3:** Hydrological Stations in the Yellow River Source Area.

Stations	Catchment area (km^2^)	Start date	Longitude (°E)	Latitude (°N)
Huangheyan	20930	Jun. 1955	98°10′	34°53′
Tangnaihai	121972	Aug. 1955	100°09′	35°30′
Damitan	5786	Aug. 1978	100°14′	35°19′
Jungong	98414	Aug. 1979	100°39′	34°42′
Jimai	450019	Jun. 1958	99°39′	33°46′
Mentang	59655	Aug. 1987	101°03′	33°46′
Tangke	5374	Sep. 1978	102°28′	33°25′
Maqu	86048	Jan. 1959	102°05′	33°58′
Ruoergai	4001	May. 1980	102°56′	33°35′

### Modelling spatial-temporal variation in the water supply using InVEST

The water yield module of InVEST (Integrated Valuation of Environmental Services and Tradeoffs) model was used to assess the water supply [35]. InVEST is a geographic information systems (GIS) based software package for ecosystem services modeling, mapping and valuation (http://www.naturalcapitalproject.org/InVEST.html). It uses maps and tabular data of land use and land management in conjunction with environmental information (e.g., soil, topography and climate) to generate spatially explicit predictions of the biophysical supply of ecosystem services. InVEST model is continually improved and the version 2.5.3 was used in this study.

The InVEST water yield module is an estimation toolbox based on water balance. It determines the level of water yield for each pixel as the precipitation minus the water fraction of evapotranspiration. The module assumes that the water yield from a pixel reaches the point of interest via one such pathway. It then sums and averages the water yields for the sub-basin level. Pixel-scale calculations were used to represent heterogeneity in the key driving factors for water yield, such as soil type, precipitation and vegetation type. Comparing to other hydrological models, the InVEST water yield module is simple and could easily be parameterized and calibrated. The key equations of InVEST water yield module were given as follows:

$$Y_{xj}=\left(1-\frac{AET_{xj}}{P_{x}}\right)\cdot P_{x}$$ (1)

where *Y*_*jx*_ is the annual water yield for the land cover type *j* in the grid cell *x* (*x*, *j* = 1, 2, 3…), *AET*_*xj*_ is the real annual evapotranspiration for pixel *x* with LULC *j*, and *P*_*x*_ is the annual precipitation (mm) for pixel *x*. The water balance evapotranspiration partition *AET*_*xj*_/*P*_*x*_ is a Budyko curve approximation developed by Zhang et al. [36]:

$$\frac{AET_{xj}}{P_{x}}=\frac{1+\omega_{x}R_{xj}}{1+\omega_{x}R_{xj}+\frac{1}{R_{xj}}}$$ (2)

where *R*_*xj*_ is the dimensionless Budyko dryness index at pixel *x* with LULC *j*, which is defined as the ratio of potential evapotranspiration to precipitation [37]. *ω*_*x*_ is the modified dimensionless ratio of plant accessible water storage to the expected precipitation during a year, which was defined by Zhang et al. [36] as a non-physical parameter that characterizes the natural climatic-soil properties:

$$\omega_{x}=Z\frac{AWC_{x}}{P_{x}}$$ (3)

where *AWC*_*x*_ is the plant available water content volume (mm). The soil texture and effective soil depth define *AWC*_*x*_, which is the amount of water that can be held and released in the soil for use by plants and is estimated as the product of the difference between field capacity and wilting point and the minimum of soil and root depths. *Z* is a seasonality factor that defines the seasonal rainfall distribution and depths. Finally, the Budyko dryness index was defined as follows, for which *R*_*xj*_ values greater than 1 denote pixels that are potentially arid [37]:

$$R_{xj}=\frac{k_{xj}\cdot ET_{0}}{P_{x}}$$ (4)

where *ET*_0*x*_ is the reference evapotranspiration at pixel *x*, and *k*_*xj*_ is the plant (vegetation) evapotranspiration coefficient associated with LULC *j* on pixel *x*. *ET*_0*x*_ represents the climatic demand approximation, while *k*_*xj*_ is primarily determined from the vegetative characteristics for pixel *x* [38].

The input variables for InVEST water yield module include average annual precipitation (*P*_*x*_), annual reference crop evapotranspiration (*ET*_0_), soil depth, plant available water content (*AWC*_*x*_), land use and land cover (LULC), root depth and elevation. We calculated the *AWC*_*x*_ (i.e., the fraction of available water that can be stored in the soil profile for plant use) based on soil texture data. *ET*_0_ was calculated using the Penman-Monteith equation. The required parameters included the Zhang coefficients and vegetation evapotranspiration coefficient (*K*_*xj*_). Details of the input variables and parameters can be found in Table 4.

**Table 4:** Input variables and parameters for InVEST water yield module.

Variables and parameters	Description and inputs
*Z*	Zhang coefficient, a seasonality factor that defines the seasonal rainfall distribution and depths, calibrated with a water balance method
*K*_*xj*_	Vegetation evapotranspiration coefficients, proposed by FAO [38]
*P*_*x*_	Annual precipitation for pixel *x* (mm), spatialization from metrological stations records using ANUSPLIN 4.2
*ET*_0_	Reference crop evapotranspiration (mm), calculated with the FAO56-modified Penman-Monteith equation [34]
*AWC*_*x*_	Plant available water content volume (mm), calculated following the methods and processes in Zhou et al. [39]
LULC *j*	Land use and land cover change, interpreted from Landsat thermic mapper/enhanced thermic mapper (TM/ETM) satellite images
Elevation	A digital elevation model (DEM) data with 90m resolution from SRTM Digital Elevation Database was used
Root depth	Root depth were collected from spatially rasterized 1:1,000,000 soil map of China
Soil depth	Soil depth were collected from spatially rasterized 1:1,000,000 soil map of China

### Quantifying the contributions of climate and land use changes

The Residual Trends (RESTREND) method was applied to distinguish the relative contributions of climate and land use change to water supply variations [40–41]. In this study, we firstly calculate the actual water supply variations during the past three decades based on the calibrated and validated InVEST model. Then, two scenarios were presumed: (1) the climate did not change with only land use signal, and (2) the land use did not change with only climate change signal. The water supplies under different scenarios were simulated by fixing the climate and land use input terms respectively in the InVEST model. After that, the residual trends will be derived from the differences between actual trends and scenarios trends. Finally, the relative contributions of climate and land use to water supply variations will be disentangled. The detailed processes are given as follows:

$$\Delta W_{T}=W_{R}-W_{B}$$ (5)

$$\Delta W_{L}=W_{R}-W_{RC}$$ (6)

$$\Delta W_{C}=W_{R}^{}-W_{RL}$$ (7)

$$\eta_{L}=\frac{\Delta W_{L}}{\Delta W_{T}}\times 100\%$$ (8)

$$\eta_{C}=\frac{\Delta W_{C}}{\Delta W_{T}}\times 100\%$$ (9)

Where, △*W*_*T*_ is the overall water supply variation in a specific period; *W*_*R*_ is the actual water supply at the end of the period; *W*_*B*_ is the baseline water supply at the beginning of the period; △*W*_*L*_ is the effects of land use change on water supply; △*W*_*RC*_ is the water supply under the scenario with only climate change; △*W*_*C*_ is the effects of climate change on water supplies; *W*_*RL*_ is the water supply under the scenario with only land use change; *η*_*L*_, *η*_*C*_ are the contributions of land use and climate change to water supplies respectively.

## Results

### Calibration and validation for InVEST water yield module

**Fig 2:**
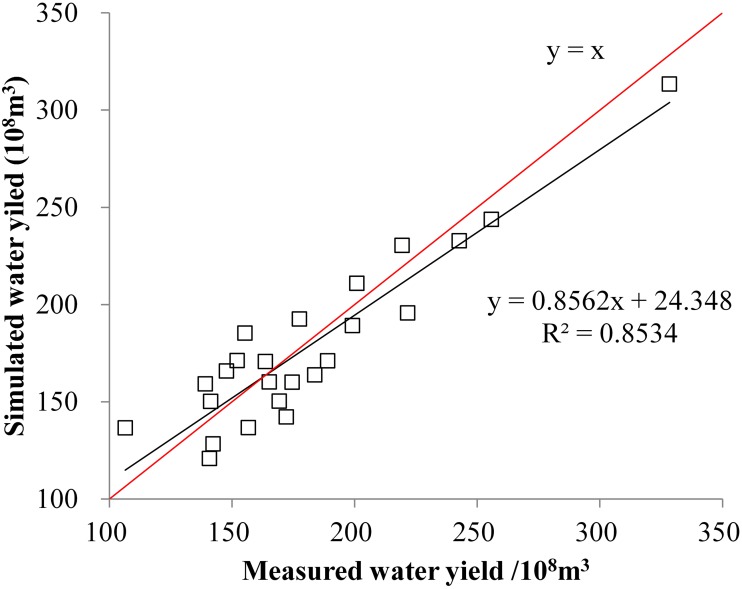
Comparison between the simulated and measured water yields in YRSA.

The InVEST water yield module is based on a simple water balance where it is assumed that all water in excess of evaporative loss arrives at the outlet of the watershed. The module is an annual average time step simulation tool applied at the pixel level but reported at the sub-basin level. A first run model calibration and validation should be performed. Before starting calibration processes, sensitivity analysis using the observed runoff data was carried out to define the parameters that influence model outputs the most. The calibration then focused on highly sensitive parameters followed by less sensitive ones. The model was calibrated and validated using hydrological records from 1985 to 2008 for the Tangnaihai Station, which is the station that controls the YRSA. The observed flow data were converted to units of m^3^/year and compared with the simulated water-yield volume. The preliminary simulation data were compared with the observation data, and we referred to relevant literature and data to fine-tune the parameters for validation. The final validation results show that the determination coefficient (*R* ^*2*^) between the simulation results and measured data is greater than 0.85 (Fig 2). The parameters determined through such validation were used for the model and results.

### Observed climate and LULC changes during past three decades

#### Annual temperature and precifpitation changes

**Fig 3:**
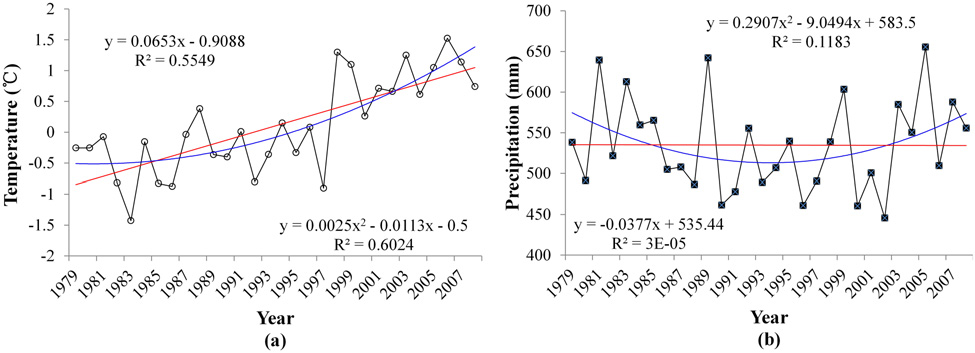
Change trends of annual mean temperature (a) and precipitation (b) in the YRSA over the past three decades.

**Fig 4:**
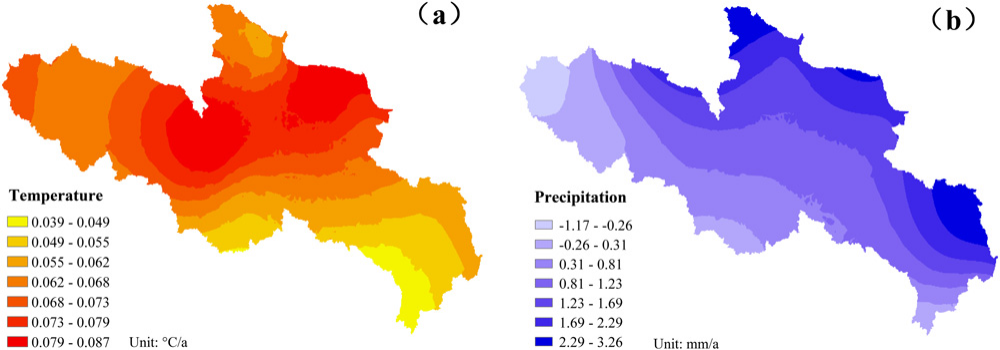
Spatial distribution of temperature (a) and precipitation (b) change rates in YRSA over the past three decades, the unit for (a) is °C/a and mm/a for (b).

The annual temperature and precipitation change trends from 1979 to 2008 were analyzed (Fig 3). A Mann-Kendall method was used to test the significance of both trends of temperature and precipitation. Over the past three decades, this area has exhibited a significant warming trend at 0.05 level with a rate of 0.0653°C/a. The mean temperature of each year from 1996 to 2008 was higher than 0°C, except for 1997. However, there were several extreme cold years, including 1983 (-1.4°C), 1992 (-0.8°C) and 1997 (-0.9°C). The hottest year was 2006, with a mean temperature of 1.5°C. Precipitation in the YASA shows a drying trend but isnot significant. The linear rate for the precipitation decrease is -0.0377 mm/a. To further analyze the characteristic of climate change in the YRSA, we also determined the spatial variability in temperature and precipitation over the past three decades (Fig 4). The rate of variation in the annual temperature for the entire YRSA has been positive for the past three decades; thus, temperature showed an upward trend. In particular, the rate of variation in the annual temperature is relatively large for the north-central area with a maximum of up to 0.087°C/a; the rate is relatively low in the southeast at approximately 0.039°C/a. The precipitation trended upward for part of the area but downward for other regions. The annual positive variation rate is relatively large for certain northern and eastern areas in the YRSA with a maximum of up to 3.26 mm/a; the annual variation rate in southern and western areas is negative reaching as low as -1.17 mm/a.

### LULC changes

**Fig 5:**
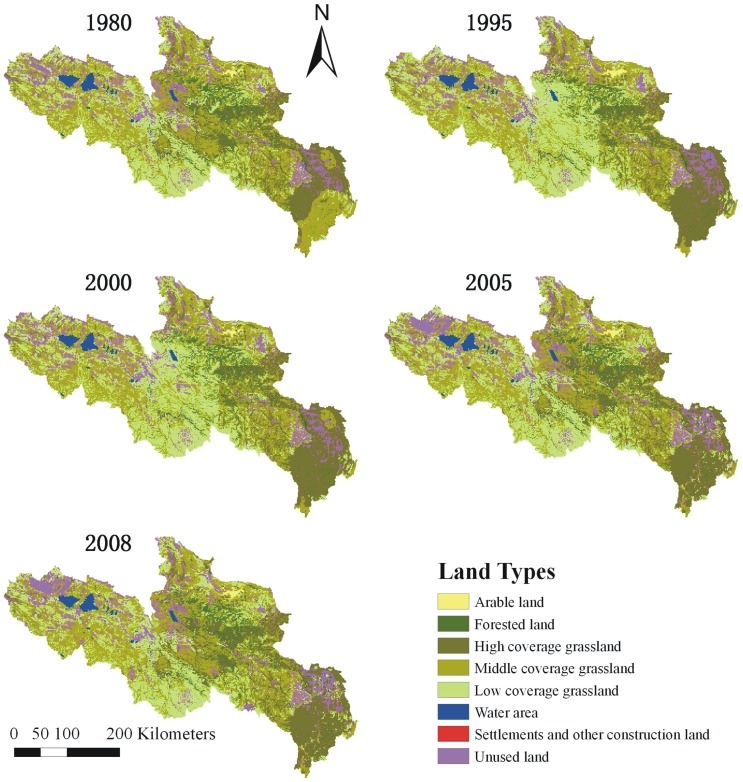
Land use and land cover change in YRSA of different periods during the past three decades.

**Fig 6:**
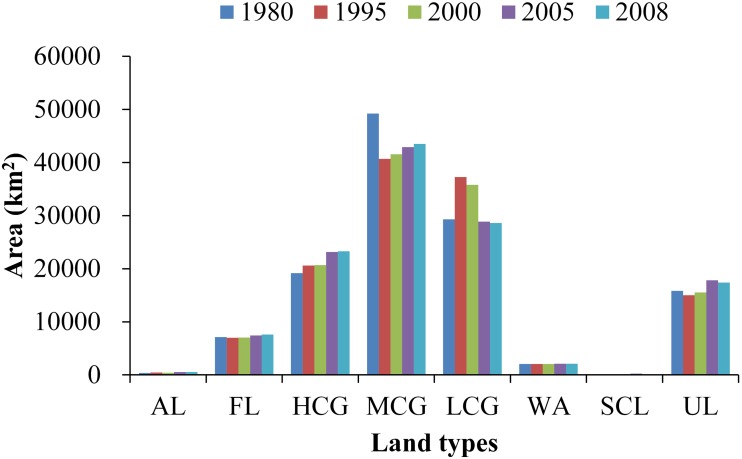
The area changes among land types of different periods in YRSA over the past three decades.

The land use structure of YRSA over the past three decades indicates no considerable changes based on the satellite images. However, the spatial and temporal variations among different LULC types were observed (Fig 5). Due to the combined constraints from multiple natural conditions, arable land in the YRSA is primarily concentrated in the river terrace and alluvial plains. From 1980 to 2008, the arable land area in the YRSA increased from 377 km^2^ in 1980 to 542 km^2^ in 2008. The forested land area slightly decreased from 7141 km^2^ in 1980 to 6996 km^2^ in 1995. However, the forested land area began to increase from 1995. The area increased to 7,030 km^2^ in 2000 and 7,412 km^2^ in 2005, which exceeded the level in 1980, and reached 7,585 km^2^ in 2008. The grassland area did not change considerably from 1980 to 1995. It substantially decreased in 2000 and then continued to decrease up to 2005 from 98,069 km^2^ to 94,915 km^2^. In 2008, the grassland area began to increase to 95,383 km^2^ due to the Three Rivers Source Area Ecological Protection Project (Fig 6). In particular, the high coverage grassland area did not change significantly. The middle coverage grassland area substantially decreased from 1980 to1995 and became low coverage grasslands or desert. It then slowly increased, which corresponded with the substantial increase in low-coverage grasslands during 1980 to 1995. The open water area did not change considerably. The settlements and construction land did not change significantly from 1980 to 2000, whereas it increased substantially in 2005 from 15 km^2^ to 249 km^2^. The unused land generally increased during 1980–2005, which was likely associated with grassland desertification, and it slightly decreased in 2008.

### Spatial-temporal variation of Water supplies changes

**Fig 7:**
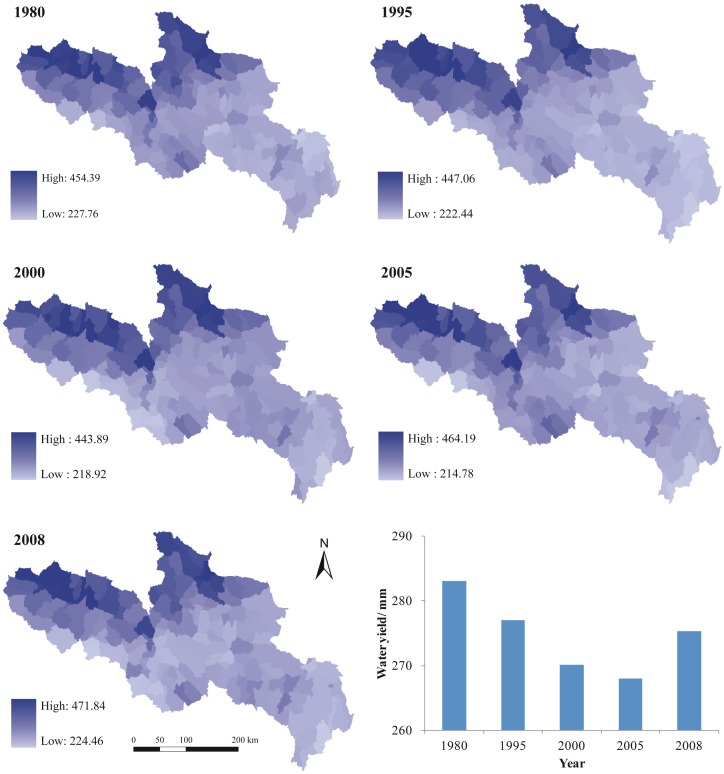
Water supplies over YRSA in different periods at sub-water scale during the past three decades, the unit is mm.

Using the InVEST model, the YRSA water supplies were assessed for different periods including 1980, 1995, 2000, 2005 and 2008 (Fig 7). The average water supply decreased slightly from 283.01 mm in 1980 to 276.95 mm in 1995, 270.12 mm in 2000 and 267.97 mm in 2005; it then rebounded slightly to 275.26 mm in 2008. The water supplies varied between 283.01 mm and 267.97 mm during the period 1975–2008, and the highest year is 1980 and lowest is 2005. The spatial pattern shows that the northwestern regions in YRSA have higher water supplies, including Qumalai, Maduo, Maqin and Xinghai Counties, while the southeastern regions have lower water supplies, including Hongyuan Ruoergai, Aba, Maqu and Jiuzhi Counties.

**Fig 8:**
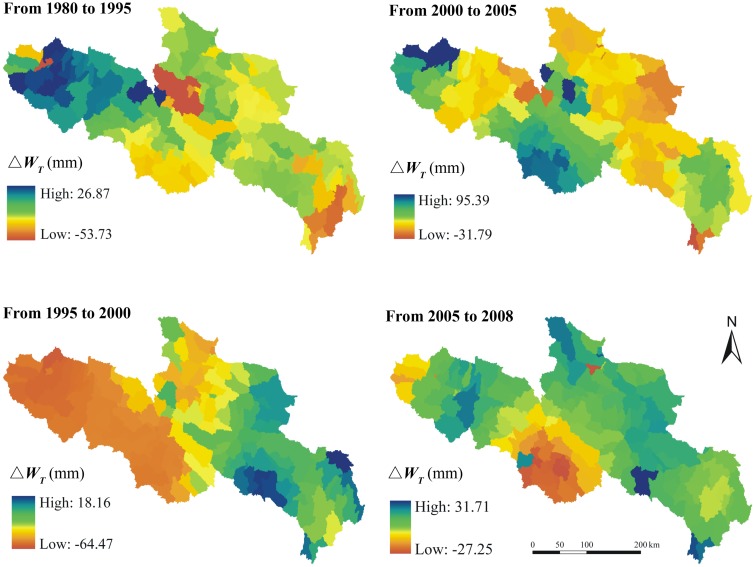
Water supplies change of different periods (△*W_T_*) over the past three decades in YRSA, the unit is mm.

To more clearly reveal the water supply variations of YRSA during different periods, we analyzed the water supply changes at different stages over the past three decades, including four stages of 1980–1995, 1995–2000, 2000–2005 and 2005–2008 (Fig 8). Although the first three stages showed an overall decreasing water supply, some specific areas indicate increasing water supplies. The regional difference of water supplies variations is obvious. From 1980 to 1995, the water supply increased in some parts of the western region, whereas the water supply significantly decreased in most other areas. Particularly, the water supply in parts of Maqin and Hongyuan Counties was 53.73 mm which is lowest. From 1995 to 2000, the regional differences pattern was typically the opposite of that observed in the previous period. Most areas in the western part show a downward trend. The water supply slightly increased in the eastern including Jiuzhi and Ruoergai Counties. However, the decreasing areas were larger than increasing areas. From 2000 to 2005, the water supply increased in Qumalai, Chengduo, Dari, Gande and Banma counties in the western and Ruoergai and Hongyuan Counties in the eastern. However, the water supply decreased in other areas. From 2005 to 2008, the water supply show an upward trend in most areas, but decreased in some specific southern areas, such as Dari County.

### RESTREND analysis

**Fig 9:**
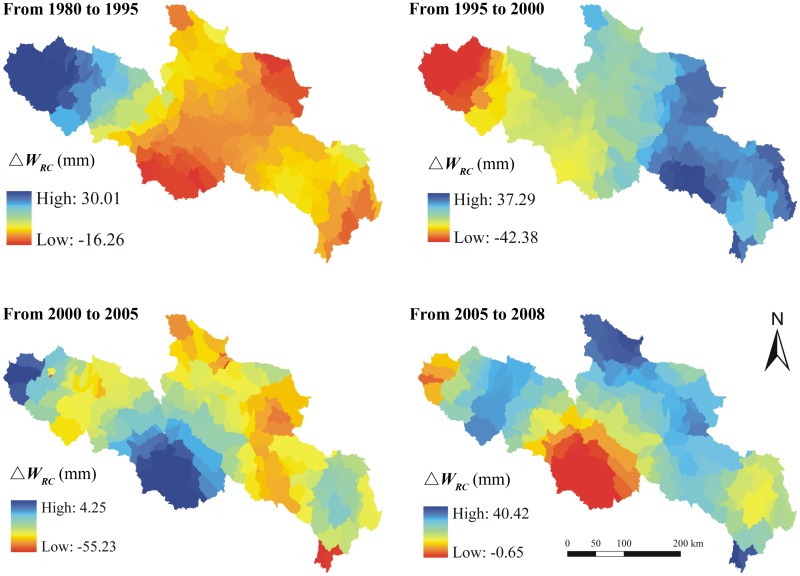
Variations of the water supply under the scenarios of no land use change (△*W_RC_*) in different periods over YRSA, the unit is mm.

**Fig 10:**
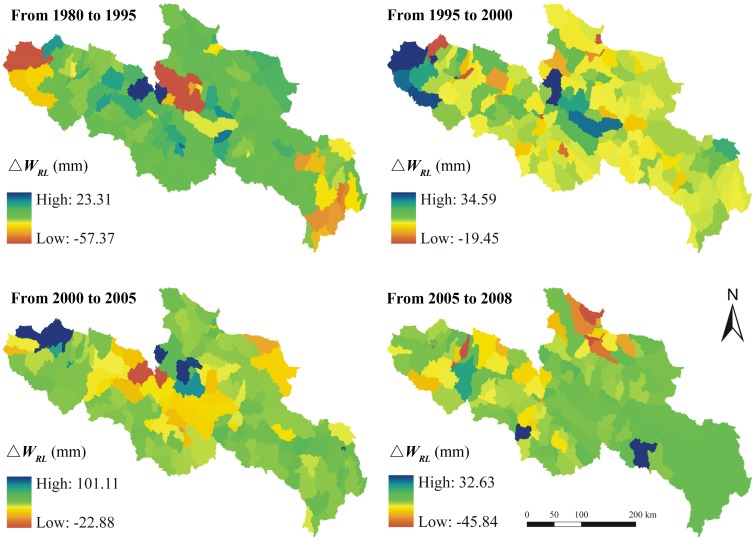
Variations of the water supply under the scenarios of no climate change (△*W_RL_*) in different periods over YRSA, the unit is mm.

RESTREND analysis was carried out to evaluate the relative contributions of climate and land use changes to water supplies in YRSA. The water supplies under two different scenarios were calculated. In the first scenario, we assumed land use did not change which means the water supply variations were mainly driven by climate change during the past three decades. Under this scenario, the water supply shows a ‘decrease-increase-decrease-increase’ trend (Fig 9). It decreases in much of YRSA from 1980 to 1995 but increases in most areas from 1995 to 2000. During the period of 2000 to 2005, the water supply decreases again in parts of the eastern and northern region. The water supply increases again in major regions of YRSA from 2005 to 2008, except for Dari County. In the other scenario, we assumed climate did not change which means the water supply variations were only driven by land use change from 1980–2008 (Fig 10). Under this scenario, the area where the water supply increases is larger than that deceases from 1980 to 1995. However, the water supply decreases in most areas from 1995 to 2000. As to 2000 to 2005, the water supply generally decreases in most areas but increases in some specific regions. From 2005 to 2008, the water supply increases in most areas.

**Fig 11:**
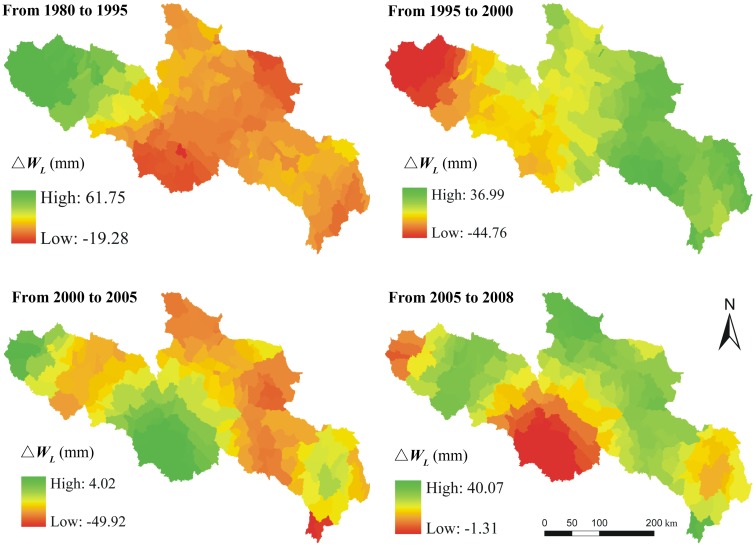
Residuals between actual water supply changes and changes due to land use (△*W_L_*) in different periods over YRSA.

**Fig 12:**
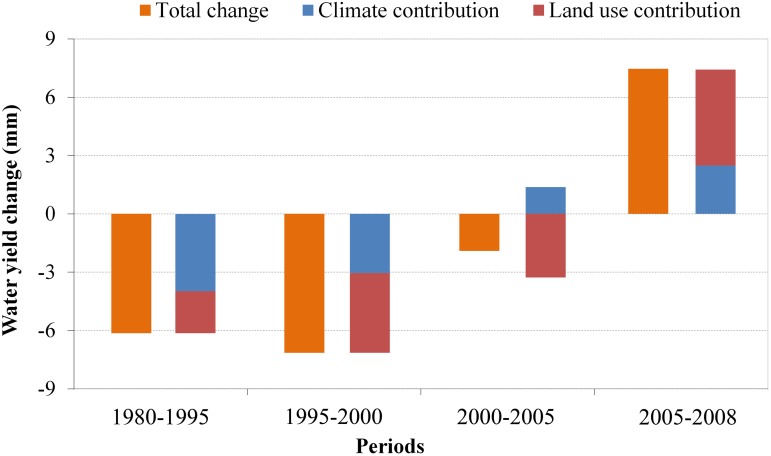
Relative contributions of climate and land use change to water supplies variations in different periods over YRSA.

To distinguish the relative contributions of climate and land use changes to water supply quantitatively, the residuals between actual and scenarios of different periods were calculated. Fig 11 shows the residuals between actual water supplies and the water supplies under the scenario with only land use change. We compared the variations of average water supplies as well as the average residuals among different periods which reflect the relative contributions from climate and land use respectively (Fig 12). Over the past three decades, the water supply kept decreasing from 1980 to 2005, most significantly from 1995 to 2000 and less significantly from 2000 to 2005. From 2005 to 2008, the water supply has greatly increased. The climate and land use changes generally had a negative impact on the YRSA water supply from 1980 to 1995 and 1995 to 2000. The negative contribution from climate change was relatively more significant during the period 1980–1995 at approximately 3.98 mm in average. But the negative contribution of climate change slightly decreased to approximately 2.87 mm during the period 1995–2000. The land use change was the main driver for the water supply decreasing during this period at about 3.96 mm. From 2000 to 2005, climate change had a positive impact on water supply due to increased precipitation, whereas land use changes continued to have a negative impact due to persistent human activity. During 2005 to 2008, both climate and land use changes had a positive impact, and the land use contribution was approximately 4.43 mm in average.

## Discussion

### Relative contributions of land use and climate changes to water supply variations

Alpine grassland is the primary ecosystem type in the area studied herein, and their water supply is primarily provided by soil water storage, litter water holding, canopy origins and surface runoff. From a water balance perspective, precipitation and actual evapotranspiration are the two critical factors that determine the water supply of an ecosystem. Previous studies have qualitatively or semi-quantitatively investigated portions of the variations and the driving mechanisms thereof in water retention or supply for the area studied herein and also adjacent areas. Wang et al. [42] constructed a water retention index for the YRSA and discussed its variation characteristics. He suggested that the serious extent of ecosystem degradation on the land surface is the dominant factor affecting the variation in the regional water cycle for hydrological processes. Nie et al. [43] preliminarily estimated the water conservation capacity for the Tibetan Plateau for the period 1982–2003 based on the principles of water balance and surface energy balance. However, they did not further quantitatively analyze the relative contributions of climate or land use change for such variations in water retention capacity.

Our work more thoroughly and further explores the individual contributions of climate and land use changes. The RESTREND analysis results show that climate change had a negative impact on most areas from 1980 to 1995 which is about 64% contribution to the total water supply change; nevertheless the impacts of land use changes were large enough in some areas to affect a significant portion of observed water supplied change in the YRSA. It is mainly because the decreasing precipitation which reduced the water sources during this period, though the forested land area has slightly decreased. The climate change also had a positive impact on few western parts including Qumalai County, Chengduo County and part of Maduo County. During 1995 to 2000, climate change contributed negatively to water supply in the western part of YRSA as the rising temperature caused the evapotranspiration increasing, but it had a positive impact on the western area. Averagely, the percentage contribution of climate change in this period was about 42%. Although the precipitation began to increase, the land use change contributed more to the water supply decreasing from 1995 to 2000 as the grasslands have been seriously degraded. As the continuous grasslands degradation from 2000 to 2005, the water supply decreased again in parts of the eastern and northern regions although the precipitation slightly increased which contributed positively to the water supply. But the positive contribution of climate change is lower than the negative contribution of land use change, so the water supply was reduced. In the last period of 2005 to 2008, the positive contribution of precipitation increasing was greater than the negative contribution of rising temperature. As a result, the climate change showed a positive impact overall. The positive impact of climate change was primarily concentrated on the western area, and the negative impact was primarily concentrated on the mid-eastern region. More important, benefited from the implementation of Three Rivers Source Area Ecological Protection Project, the vegetation coverage conditions have greatly improved which raised the water retention ability of this region. As a result, both the land use and climate change contribute positively to the water supply increasing from 2005 to 2008. But the contribution of land use is about 60.8% which is higher than climate change.

### Uncertainties in this study

Firstly, after calibrating the parameters, the water yield module of InVEST model reasonably captures the temporal and spatial patterns of water yields over YRSA. However, the module is based on a simple water balance principle, and it assumes that the water produced in a watershed in excess of the evapotranspirative loss arrives at the watershed outlet, without considering water capture by means other than primary human consumptive uses [35]. But the relative contribution of yield from various parts of the watershed should still be valid. The module is also an annual average time-step simulation tool applied at the pixel level but reported at the sub-basin level, which neglect extremes and do not consider the temporal dimensions of water supply [44]. It does not consider sub-annual patterns of water delivery timing. Thus, the ability of mechanism explanation for InVEST model is relatively weak [45]. It is difficult to explain complex hydrological processes using this model. In this study, our goal is to reveal the water supplies changes and the relative contributions of land use and climate change. We don’t want to pay much attention to the detailed processes and mechanisms of hydrological processes though it is very important. So, we believed that the InVEST model is proper for this study. Nevertheless, further works on parameters calibration and model validation are necessary. Alternatively, more models with different structures should be used to represent the uncertainties from models. Secondly, although we tried to collect more accurate data, certain uncertainties in the data are existed as observation errors and different data processing methods applied. For example, certain errors may exist in the processes of climate data spatialization as the meteorological stations are relatively sparse in plateau area. Another uncertainty in the data is the remote sensing based land use and land cover data. Although we divided the grassland into three different land types including high coverage, middle coverage and low coverage grassland, it is hard to detect the actual grassland degradation only based on remote sensed data. The grassland degradation is not only vegetation coverage decreasing but also the changes of plants population etc. which is hard to detect by satellites. In addition, there are interactions between LUCC and climate change which are not considered in this study. It is generally believed that human activities through land use are the primary causes of the land cover change over a relatively short temporal scale such as three decades, while the climate change has little impacts [46]. In the other hand, landscape changes could alter large-scale atmospheric circulation patterns far from where the land use and land cover changes occur [47]. It has less impact on local climate. Overall, these uncertainties will not affect the results obtained and conclusions drawn herein. But this study could be further improved by accounting for such uncertainties.

## Conclusions

Recent climate and LULC trends produce a discernible impact on simulated water supply over the Yellow River Source Area (YRSA) in Tibetan Plateau during past three decades. The detailed meteorological, hydrological records and satellite data over YRSA from 1980s to 2008, together with the InVEST water-yield module and RESTREND method, were used to assess the water supplies change over the past three decades and discriminate the relative contributions from climate and land use changes to water supply variations.

The water supply significantly decreased from 1980 to 2005 and then increased from 2005 to 2008 in the YRSA. The contributions of climate and land use changes varied among different periods over the past three decades. From 1980 to 1995, climate change contributed dominantly to water supply decreasing with a about 64% contribution. Land use contributed more than climate change to the water supply decreasing with a roughly 58% contribution as the intense human activities from 1995 to 2005. Began from 2000, the climate change became a positive contribution to the water supply as the increased precipitation in this period, but the land use still contributed negatively. From 2005 to 2008, both climate and land use have a positive impact on the water supply, but the contribution of land use change is about 60% which is higher than climate change. The implementation of Three Rivers Source Area Ecological Protection Project has greatly improved the vegetation coverage conditions and the water retention ability. The conclusions of this study could provide a scientific basis for ecosystem restoration construction, climate-change adaptation and land use management in YRSA. Ecological protection projects, grazing policies, artificial improvement of degraded grassland etc. would help to conserve the water retention ability and increase water supply over this region.

## References

[1] Field CB, Barros VR, Dokken DJ, Mach KJ, Mastrandrea MD, Bilir TE, et al (2014) IPCC, 2014: Summary for policymakers. In: Climate Change 2014: Impacts, Adaptation, and Vulnerability. Part A: Global and Sectoral Aspects Contribution of Working Group II to the Fifth Assessment Report of the Intergovernmental Panel on Climate Change. Cambridge University Press, Cambridge, United Kingdom and New York, NY, USA, pp. 1–32.

[2] Daily GC, Matson PA (2010) cosystem services: From theory to implementation. Proceedings of the National Academy of Sciences of the United States of America, 105(28): 9455–9456. 10.1073/pnas.0804960105 18621697 PMC2474530

[3] Millennium Ecosystem Assessment (2005) Ecosystems and Human Well- Being: Synthesis. Washington, DC: Island Press.

[4] Allan JD, McIntyre PB, Smith SDP, Halpern BS, Boyer GL Buchsbaum A, et al (2013). Joint analysis of stressors and ecosystem services to enhance restoration effectiveness. Proceedings of the National Academy of Sciences of the United States of America 110(1): 372–377. 10.1073/pnas.1213841110 23248308 PMC3538252

[5] Su CH, Fu BJ, He CS, Lü YH (2012) Variation of ecosystem services and human activities: a case study in the Yanhe Watershed of China. Acta Oecologica 44: 6–7.

[6] Minville M, Brissette F, Leconte R (2010) Uncertainty of the impact of climate change on the hydrology of a nordic watershed. Journal of Hydrology 358(1–2): 70–83.

[7] Shi Y, Wang RS, Huang JL, Yang WR (2012) An analysis of the spatial and temporal changes in Chinese terrestrial ecosystem service functions. Chinese Science Bulletin 57(19): 2120–2131.

[8] World Commission on Dams (2000) Dams and development: A new framework for decisionmaking The Report of the World Commission on Dams. Earthscan Publications LTD, London.

[9] Ennaanay, Driss (2006) Impacts of Land Use Changes on the Hydrologic Regime in the Minnesota River Basin. Ph.D. thesis, graduate School, University of Minnesota.

[10] Su CH, Fu BJ (2013) Evolution of ecosystem services in the Chinese Loess Plateau under climatic and land use changes. Global and Planetary Change 101: 119–128.

[11] Lawler JJ, Lewis DJ, Nelson E (2014) Projected land-use change impacts on ecosystem services in the United States. Proceedings of the National Academy of Sciences of the United States of America 111(20): 7492–7497. 10.1073/pnas.1405557111 24799685 PMC4034212

[12] Lorencová E, Frélichová J, Nelson E, Vačkář D (2013) Past and future impacts of land use and climate change on agricultural ecosystem services in the Czech Republic. Land Use Policy 33: 183–194.

[13] Polasky S, Nelson E, Pennington D, Johnson K (2011) The Impact of Land-Use Change on Ecosystem Services, Biodiversity and Returns to Landowners: A Case Study in the State of Minnesota. Environmental & Resource Economics 48(2): 219–242.

[14] Nelson EJ, Kareiva P, Ruckelshaus M, Arkema K, Geller G, Girvetz E, et al (2013) Climate change's impact on key ecosystem services and the human well-being they support in the US. Frontiers in Ecology and the Environment 11(9): 483–493.

[15] Cowling RM, Egoh B, Knight AT. (2010) An operational model for mainstreaming ecosystem services for implementation. Proceedings of the National Academy of Sciences of the United States of America 105(28): 9483–9488. 10.1073/pnas.0706559105 PMC2474485 18621695

[16] Fu B, Liu Y, Lu Y, He C, Zeng Y, Wu B. (2011) Assessing the soil erosion control service of ecosystems change in the Loess Plateau of China. Ecological Complexity 8:284–293.

[17] Kremen C (2005) Managing ecosystem services: what do we need to know about their ecology? Ecology Letters 8(5): 468–479. 10.1111/j.1461-0248.2005.00751.x 21352450

[18] Tallis HM, Kareiva P (2006) Shaping global environmental decisions using socioecological models. Trends in Ecology and Evolution 21:562–568. 16876906 10.1016/j.tree.2006.07.009

[19] Carpenter SR, Mooney HA, Agard J, Doris Capistrano, Ruth S. DeFries, Sandra Díaz, et al (2009) Science for managing ecosystem services: Beyond the Millennium Ecosystem Assessment. Proceedings of the National Academy of Sciences of the United States of America, 106(5): 1305–1312. 10.1073/pnas.0808772106 19179280 PMC2635788

[20] Lü Y, Fu B, Feng X, Zeng Y, Liu Y, Chang R, et al (2012) A Policy-Driven Large Scale Ecological Restoration: Quantifying Ecosystem Services Changes in the Loess Plateau of China. PLoS ONE, 7(2): e31782 10.1371/journal.pone.0031782 22359628 PMC3280995

[21] Guo ZW, Xiao XM, Li DM (2000) An assessment of ecosystem services: water flow regulation and hydroelectric power production. Ecological Applications 10(3): 925–936.

[22] Brauman KA, Daily GC, Duarte TK, Mooney HA (2007) The nature and value of ecosystem services highlighting hydrologic services. The Annual Review of Environment and Resources 32: 67–98.

[23] Zhou GY, Wei XH, Luo Y, Zhang MF, Li YL, Qiao YN, et al (2010) Forest recovery and river discharge at the regional scale of Guangdong Province, China. Water Resources Research 46, W09503.

[24] Chen L, Xie GD, Zhang CS, Pei S, Fan N, Ge L, et al (2011)Modelling ecosystem water supply services across the Lancang River Basin. Journal of Resources and Ecology 2(4): 322–327.

[25] Zhang CQ, Li WH, Zhang B, Liu MC (2012) Water Yield of Xitiaoxi River Basin Based on InVEST Modeling. Journal of Resources and Ecology 3(1):50–54.

[26] Li Z, Liu WZ, Zhang XC, Zheng FL (2009) Impacts of land use and climate changes on hydrology in an agricultural catchment on the Loess Plateau of China. Journal of Hydrology 377(1–2): 35–42.

[27] Salhab J, Wang J, Anjum SA, Chen Y (2010) Assessment of the grassland degradation in the southeastern part of the source region of the Yellow River from 1994 to 2001. Journal of Food Agriculture and Environment 8(3–4): 1367–1372.

[28] Wen L, Dong S, Li Y, Li X, Shi J, Wang Y, et al (2013) Effect of Degradation Intensity on Grassland Ecosystem Services in the Alpine Region of Qinghai-Tibetan Plateau, China. PLOS ONE, 8(3): e58432 10.1371/journal.pone.0058432 23469278 PMC3587591

[29] Liu JY, Xu XL, Shao QQ (2010) Grassland degradation in the "Three-River Headwaters" region, Qinghai Province. Journal of Geographical Sciences 18(3): 259–273.

[30] Zhang YY, Zhang SF, Zhai XY, Xia J, et al (2012) Runoff variation and its response to climate change in the Three Rivers Source Region. Journal of Geographical Sciences 22(5): 781–794.

[31] Wang GX, Li SN, Hu HC, Li YS, et al (2009) Water regime shifts in the active soil layer of the Qinghai-Tibet Plateau permafrost region, under different levels of vegetation. Geoderma, 149(3–4): 280–289.

[32] Hutchinson MF, Gessler PE (1994) Splines: More than just as moothinterpolator. Geoderma, 62: 45–67.

[33] Hutchinson MF (1991) The application of thin plate smoothing splines to continent-wide data assimilation In: Jasper JD (eds.) BMRC Research Report No.27, Data Assimilation Systems. Melbourne: Bureau of Meteorology, 104–113.

[34] Yin YH, Wu SH, Zheng D, Yang QY, et al (2010) Radiation calibration of FAO56 Penman-Monteith model to estimate reference crop evapotranspiration in China. Agricultural Water Management 95: 77–84

[35] Tallis HT, Ricketts T, Guerry AD, Wood SA, Sharp R, Nelson E, et al (2013) InVEST 2.5.3 User’s Guide The Natural Capital Project, Stanford.

[36] Budyko MI (1974) Climate and Life, Academic, San Diego, California.

[37] Zhang L, Dawes WR, Walker GR (2001) Response of mean annual evapotranspiration to vegetation changes at catchment scale. Water Resources Research 37:701–708.

[38] Allen RG, Pereira LS, Raes D, Smith M (1998) Crop Evapotranspiration: Guidelines for computing crop water requirement FAO Irrigation and Drainage paper 56. Rome: United Nations Food and Agriculture Organization.

[39] Zhou WZ, Liu GH, Pan JJ, Feng XF (2005) Distribution of available soil water capacity in China. Journal of Geographical Sciences 15(1): 3–12.

[40] Evans J, Geerken R (2004) Discriminating between climate and human-induced dryland degradation. Journal of Arid Environments 57: 535–554.

[41] Wessels KJ, Prince SD, Malherbe J, Smallc J, Frostd PE, VanZylb D (2007) Can human-induced land degradation be distinguished from the effects of rainfall variability? A case study in South Africa. Journal of Arid Environments 68: 271–297.

[42] Wang GX, Liu GS, Li CJ, Yang Y (2012) The variability of soil thermal and hydrological dynamics with vegetation cover in a permafrost region. Agricultural and Forest Meteorology 162: 44–57.

[43] Nie YH, Gong B, Li Z (2010) The spatial temporal variations of water conservation capacity in Qinghai Tibet Plateau. Earth Science Frontiers 17(1): 373–377. (in Chinese with English abstract).

[44] Hamel P, Guswa AJ (2014) Uncertainty analysis of a spatially-explicit annual water-balance model: case study of the Cape Fear catchment, NC. Hydrology and Earth System Sciences, 11: 11001–11036.

[45] Sánchez-Canalesa M, López Benitoa A, Passuellob A, Terradoc M, Zivd G, Acuñac V, et al (2013) Sensitivity analysis of ecosystem service valuation in a Mediterranean watershed. Science of the Total Environment, 440: 140–153. 10.1016/j.scitotenv.2012.07.071 22925484

[46] Bellard C, Bertelsmeier C, Leadley P, Thuiller W, Courchamp F (2012) Impacts of climate change on the future of biodiversity. Ecology Letters, 15(4): 365–377. 22257223 10.1111/j.1461-0248.2011.01736.x PMC3880584

[47] Pielke RA Sr, Pitman A, Niyogi D, Mahmood R, McAlpine C, Hossain F, et al (2011) Land use/land cover changes and climate: modeling analysis and observational evidence. Climate Change 2011, 2:828–850.

